# Prognostic Value of a New Tool (the 3D/3D+) for Predicting 30-Day Mortality in Emergency Department Patients Aged 75 Years and Older

**DOI:** 10.3390/jcm12206469

**Published:** 2023-10-11

**Authors:** Dolors Garcia-Pérez, Anabelén Vena-Martínez, Laura Robles-Perea, Teresa Roselló-Padullés, Joan Espaulella-Panicot, Anna Arnau

**Affiliations:** 1 Emergency Service, Althaia Xarxa Assistencial Universitària de Manresa, 08243 Manresa, Spain; 2 Doctoral Program in Medicine and Biomedical Sciences, Universitat de Vic-Universitat Central de Catalunya (UVIC-UCC), 08500 Vic, Spain; 3 Faculty of Medicine, Universitat de Vic-Central de Catalunya (UVIC-UCC), 08500 Vic, Spain; jespaulella@hsc.chv.cat (J.E.-P.); aarnau@althaia.cat (A.A.); 4 Central Catalonia Chronicity Research Group (C3RG), Institut de Recerca i Innovació en Ciències de la Vida i de la Salut a la Catalunya Central (IRIS-CC), 08500 Vic, Spain; 5 UFISS Geriatric Emergncy Service, Hospital Universitari de Santa Maria de Lleida, 25008 Lleida, Spain; anav@ggss.cat; 6 Emergency Service, Hospital de Figueres (Fundació Salut Empordà), 17600 Figueres, Spain; lrobles@salutemporda.cat; 7 Althaia Xarxa Assistencial Universitària de Manresa, 08243 Manresa, Spain; trosello@althaia.cat; 8 Geriatric and Palliative Care Service, Consorci Hospitalari Vic, Fundació Hospital de la Santa Creu de Vic, 08500 Vic, Spain; 9 Research and Innovation Unit, Althaia Xarxa Assistencial Universitària de Manresa, 08243 Manresa, Spain

**Keywords:** emergency department, elderly, geriatric assessment, frailty, frailty transitions, mortality, clinical frailty scale, identification of seniors at risk screening tool

## Abstract

The 3D/3D+ multidimensional geriatric assessment tool provides an optimal model of emergency care for patients aged 75 and over who attend the Emergency Department (ED). The baseline, or static, component (3D) stratifies the degree of frailty prior to the acute illness, while the current, or dynamic, component (3D+) assesses the multidimensional impact caused by the acute illness and helps to guide the choice of care facility for patients upon their discharge from the ED. The objective of this study was to evaluate the prognostic value of the 3D/3D+ to predict short- and long-term adverse outcomes in ED patients aged 75 years and older. Multivariable logistic regression models were used to identify the predictors of mortality 30 days after 3D/3D+ assessment. Two hundred and seventy-eight patients (59.7% women) with a median age of 86 years (interquartile range: 83–90) were analyzed. According to the baseline component (3D), 83.1% (95% CI: 78.2–87.3) presented some degree of frailty. The current component (3D+) presented alterations in 60.1% (95% CI: 54.1–65.9). The choice of care facility at ED discharge indicated by the 3D/3D+ was considered appropriate in 96.4% (95% CI: 93.0–98.0). Thirty-day all-cause mortality was 19.4%. Delirium and functional decline were the dimensions on the 3D/3D+ that were independently associated with 30-day mortality. These two dimensions had an area under receiver operating characteristic of 0.80 (95% CI: 0.73–0.86) for predicting 30-day mortality. The 3D/3D+ tool enhances the provision of comprehensive care by ED professionals, guides them in the choice of patients’ discharge destination, and has a prognostic validity that serves to establish future therapeutic objectives.

## 1. Introduction

Emergency care in older adults is a public health problem that needs to be addressed. The progressive aging of the population is accompanied by an increase in emergency visits and hospital admissions among the elderly. This subgroup constitutes a highly heterogeneous population characterized by different ways of aging, frailty, multimorbidity, polypharmacy and often atypical presentations of diseases [1,2]. The current *modus operandi* in place at emergency departments (ED) is highly protocolized, focused on the diagnosis–treatment–survival of the patient in order to guarantee quality of care [3]. This may explain why emergency physicians attending to older adults tend to take a short medical history and center on the reason for the consultation. Caring for these patients is resource-intensive and frequently requires admission to the hospital, in spite of the significant risk that hospitalization entails in these cases [4].

There is currently a broad consensus among the European emergency and geriatric societies to the effect that attempts to detect and monitor frailty among elderly patients in the ED should be prioritized in order to improve health results. Frailty is a clinical entity that is strongly associated with age, characterized by a loss of strength and resistance and reduced physiological functions, which increases individual vulnerability and affects the ability to cope with different stress situations [5,6,7]. Frailty is dynamic and reversible; its detection is essential in the ED, since together with the degree of severity of the acute illness, it is correlated with worsened states of health and a higher risk of mortality [6,8]. Therefore, frailty is an important factor in clinical decision making and in the design of individualized patient care [9].

The most widely validated instrument in clinical practice for the diagnosis of frailty, and the one with the greatest evidence of benefit, is the Comprehensive Geriatric Assessment (CGA) [10,11]. Its aim is to exhaustively identify clinical, functional, cognitive and social problems in order to design the best possible therapeutic strategy from the point of view of efficacy and resource optimization. However, the CGA cannot be used in the ED. Several authors have presented brief, simple scales such as the Identification of Seniors at Risk screening tool (ISAR) [12] and the modified Rockwood Scale or Clinical Frailty Scale (CFS) [13,14] for the identification of frail elderly in the ED. Despite their ease of application and their ability to predict poor health outcomes in the short–medium term, their use has not become standardized in EDs in Spain [15]. However, the assessment of frailty is an essential element of clinical care of the elderly in EDs, given its key role in decision making and in the proposal of emergency care plans [16].

Here, we describe a new express geriatric assessment tool, the 3D/3D+ [17], developed and validated at our ED. This tool is based on elements of the CGA, and the domains that comprise it were selected by consensus among the referring GPs and geriatricians who are members of the working group on elderly emergency patients of the Catalan Society of Emergency Care. The 3D/3D+ meets the requirements defined in previous studies for an instrument to define frailty and stratify the elderly population in the ED setting [18]. It can be universally applied in the older adult population, and its brevity means that it is well suited to the dynamics of the ED. It is well calibrated for different levels of disability and useful for adapting healthcare resources. Compared with other screening instruments, the 3D/3D+ presents the novelty of allowing the simultaneous evaluation of the situational diagnosis of frailty prior to the acute illness (3D: the baseline, or static, assessment) and of the repercussion of the acute illness on the patient’s functional, cognitive, and social dimensions (3D+: the current, or dynamic, assessment). The 3D/3D+ aids decision making regarding the destination of patients on discharge from the ED, based on their degree of frailty and their need for hospital care; it may, thus, make a further contribution by proposing alternatives for elderly patients that avoid admission to acute care facilities and help to reduce the burden on the health system.

Thus, the objective of this study was to determine the prognostic value of the 3D/3D+ tool for predicting 30-day mortality and other adverse outcomes in emergency department patients aged 75 years old and over, and to compare its performance with the CFS and the ISAR.

## 2. Materials and Methods

This was a retrospective, hospital-based, single-center, observational cohort study with a 12-month follow-up. The study was approved by the reference Clinical Research Ethics Committee (CEI 20/47). The need for informed consent was waived.

The study was conducted in the ED of an acute care hospital. The Spanish National Health System is publicly funded, universal and free. It is a decentralized service and dependent on the Autonomous Communities. EDs provide care 24 h a day, 365 days, to patients who require immediate attention. The staff are trained for this care but do not have specific specialties, a situation that may lead to variations in the care provided at different EDs. Our ED has a reference population of 260,000 inhabitants. The department treats an average of 113,512 episodes per year, and patients aged 75 or over represent 20% of all emergencies. The evaluation and inclusion of the patients was carried out consecutively between 1 November 2018 and 6 January 2019. The cohort comprised all patients aged 75 or over identified as chronic complex patients (Catalan acronym PCC), or having advanced chronic disease (Catalan acronym MACA) according to the criteria of the Catalan Health Department [19,20], and also readmitted patients (with two or more hospitalizations in the last year) attending in the medical area of the ED. Patients requiring urgent surgery due to the reason for consultation were excluded.

**Figure 1:**
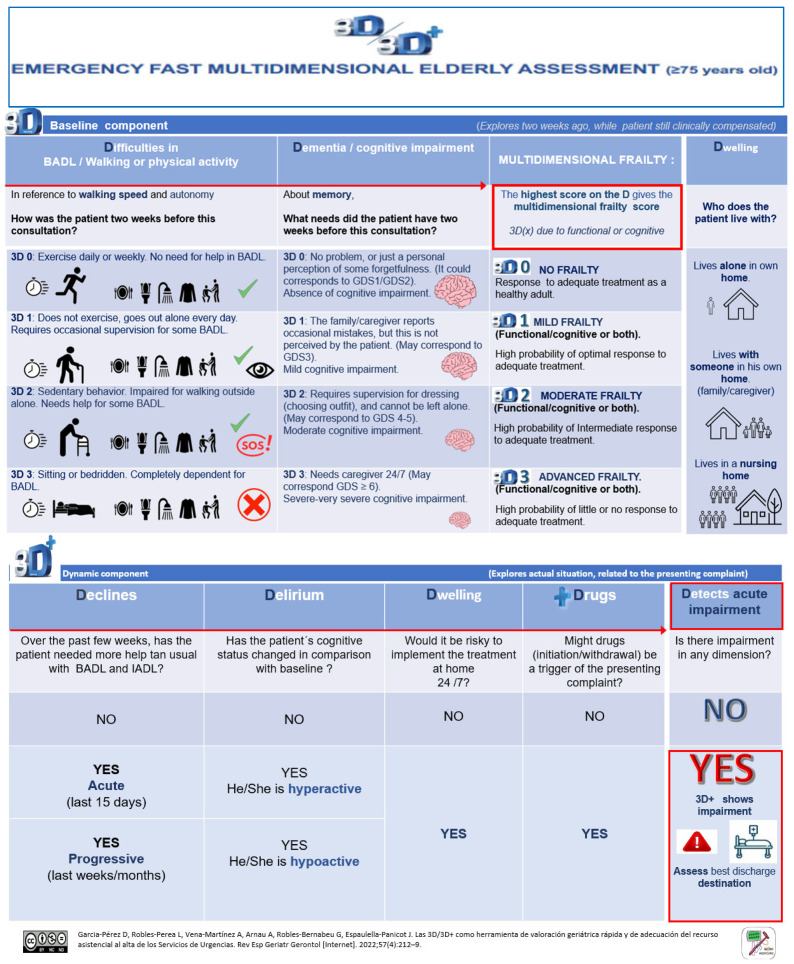
Description of the 3D/3D+ geriatric assessment tool.

During the anamnesis, the emergency physician assessed the degree of frailty of the patient using the 3D/3D+, the CFS and the ISAR scales. The 3D/3D+ is an express, mnemonic, dynamic and screening geriatric assessment tool. The 3D/3D+ records data obtained via an interview with the patient and/or caregiver, their clinical history, examination, and the physician’s clinical judgment. Drawing on this information and the clinical diagnosis, the physician is able to decide on the most suitable destination (i.e., a particular health facility or the patient’s home) upon discharge from the ED. The 3D/3D+ allows for a multidimensional assessment with seven short questions addressed to the patient/main caregiver. The 3D assessment (the baseline component) collects information on the patient’s situation over the two weeks prior to the ED visit. Three of the questions assess functional dependence, based on walking speed and autonomy in basic activities of daily living (BADL), cognitive impairment/dementia, and living arrangements. The 3D allows the categorization of patients into four multidimensional frailty profiles: no frailty (3D 0), mild frailty (3D 1), moderate frailty (3D 2) and advanced frailty (3D 3). The dimension most affected between walking/autonomy for BADL and cognitive impairment/dementia is weighted. The 3D+ assessment (the current component) explores the multidimensional repercussions of the acute illness through four questions, as a dynamic approximation of frailty taking into account the situation in which the patient lives and the medication prescribed. The 3D+ indicates the presence of acute functional decline, delirium (hyper or hypoactivity) and whether the treatment prescribed in the ED can be administered at home 24 h a day. The + serves as a reminder to ask about the drug as a possible promoter of the acute process. The 3D+ result is considered to be altered if the answer to one or more of these four questions is affirmative (Figure 1).

**Figure 2:**
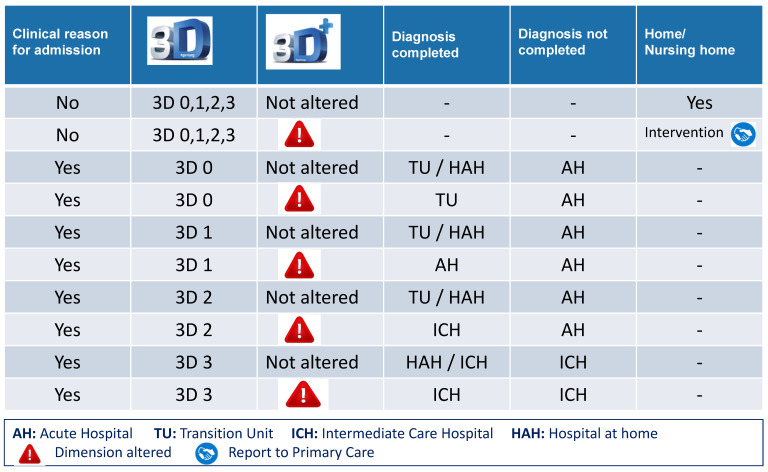
Appropriateness of the choice of the care facility on discharge from the ED, based on clinical judgement and the 3D/3D+.

**Figure 3:**
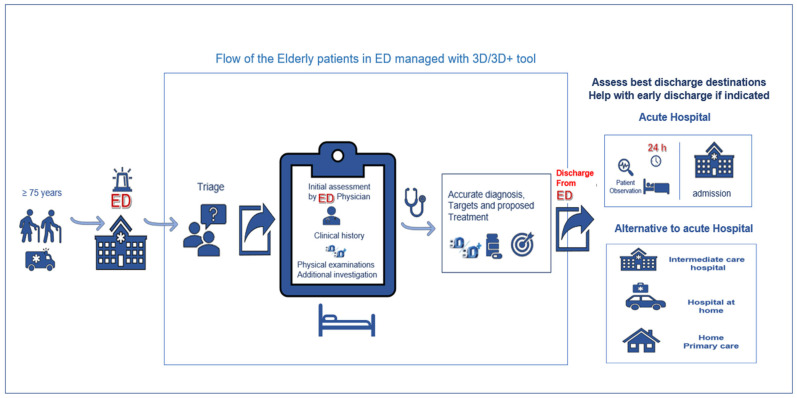
Application of the 3D/3D+ geriatric assessment tool at the Emergency Department (ED). Flowchart.

Once the clinical process is completed (anamnesis, exploration, complementary tests, diagnosis and the initiation of treatment), the 3D/3D+ assessment is used to decide on the most appropriate destination for the patient on discharge from the ED. Patients who do not require hospital admission according to their clinical diagnosis (i.e., non-severe illness, hemodynamic stability, no need for further complementary tests and eligible for treatment outside hospital) and do not present 3D+ alterations are discharged either to their own home or to a nursing home. If a dimension of the 3D+ presents an alteration, a specific intervention for this situation is required. If it cannot be resolved in the ED, the patient may be admitted to the Transition Unit, a short-stay unit for patients in this age group. If for clinical reasons hospital admission is required, based on the 3D/3D+ and the diagnosis made in the ED (i.e., no strict analytical follow-up or complementary tests are required, nor intervention by other specialists), a decision is taken regarding the most appropriate care resource: either the Transition Unit, hospital at home, standard hospitalization, the acute geriatric care unit or the intermediate care unit (Figure 2). A flowchart of the 3D/3D+ geriatric assessment is shown in Figure 3.

The variables included in this study were recorded after reviewing the emergency department report and the electronic medical record. The questionnaire prepared for the study covered demographic variables (age, sex), functional status (Barthel Index [21,22]) and cognitive status (Pfeiffer questionnaire [23]) prior to the acute illness; data on the emergency episode (time of first medical assistance in the ED, means of arrival, home medical assistance prior to the ED visit, destination on discharge from the ED); clinical variables (triage level according to the Andorran triage model, MAT-SET [24], reason for consultation, 3D/3D+, CFS score, ISAR score, main diagnosis on discharge from the ED); the preference for home care; and follow-up (repeat visit to the ED at 72 h and 30 days, admission at 72 h and 30 days, mortality at 30 days and six and 12 months). The ED physicians were responsible for the data abstraction process. The REDCap web application hosted on our institution’s server was used to enter and manage the data [25].

The main dependent variable was the prognostic accuracy of the 3D/3D+ tool for predicting 30-day mortality. The secondary dependent variables were its prognostic accuracy for predicting short-term adverse outcomes (repeat visits to the ED at 72 h and 30 days, admission at 72 h and 30 days and 30-day adverse outcome) and long-term adverse outcomes (mortality at 6 and 12 months).

The estimated sample size for a frailty prevalence of 56.6% in emergency department patients aged 75 or over according to the CFS, with a confidence level of 95% and a precision of 6%, was 263 patients. In our previous study, a total sample of 278 patients were recruited, of whom 54 (19.4%) died within 30 days of discharge from the ED [17]. This sample allowed us to obtain a two-sided 95% confidence interval with a width of 0.16 when the estimated sample AUC was 0.75.

Categorical variables are shown as absolute values and relative frequencies. Continuous variables with a normal distribution are presented as means and standard deviations, and otherwise as medians together with the 25th and 75th percentiles. The normality of the continuous variables was evaluated with the Kolmogorov–Smirnov test.

For the bivariate analysis, Student’s *t*-test was used to compare means with a normal distribution and the non-parametric Mann–Whitney U test for continuous variables with a non-normal distribution. For the comparison of qualitative variables, the chi-square test, Fisher’s exact test or the Monte Carlo exact method were used in 2 × 2 or n × 2 contingency tables in which the expected frequencies were less than 5.

Multivariable logistic regression models were used to identify the independent prognostic factors of 3D/3D+ mortality at 30 days. Four models were derived. Model 1 included the categorization of frailty according to 3D (baseline component), and model 2 included 3D and the four questions of the 3D+ assessment (current component). Model 3 included the four questions of the 3D+ assessment (current component), and in model 4 the questions of the 3D+ assessment (current component) were maintained with a value of *p* < 0.05. Adjusted odds ratios (aOR) with 95% confidence intervals (95% CI) were calculated (see Supplementary Table S1).

The aOR values from model 4 were used to calculate the risk score. The prognostic accuracy parameters (sensitivity, specificity, positive predictive value (PPV), and negative predictive value (NPV)) were estimated together with the 95% CIs of the 3D+, CFS, and ISAR. The discriminative ability of 3D+, CFS and ISAR were determined by the area under the curve (AUC) of the receiver operating characteristic (ROC), together with its 95% CI.

A two-tailed *p* value < 0.05 was considered statistically significant. For the statistical analysis, the program IBM^®^ SPSS^®^ Statistics v.29 (IBM Corporation, Armonk, NY, USA) and R^®^ version 3.3.2 (R Foundation for Statistical Computing, Vienna, Austria) were used.

## 3. Results

A total of 326 patients were evaluated for inclusion in the study, of whom 21 were excluded because they were under 75 years of age and 27 because they were not identified as PCC, MACA, or readmissions.

Thus, 278 patients were eventually included. The median age was 86 years (min–max: 75–99) and 59.7% were women. One hundred and eighty-six (66.9%) were PCC and 36 (12.9%) were MACA, and 108 (38.9%) had been admitted on two or more occasions in the last year. One hundred and nineteen (42.8%) had received previous medical care at home and 223 (80.2%) had arrived at the hospital by ambulance. One hundred and seventy-five (62.9%) were classified as priority level III by the MAT-SET. The most frequent diagnoses at discharge were cardiopulmonary diseases (109, 39.2%) and infections (72, 25.8%). One hundred and fifty-five (55.8%) presented a moderate–severe dependence for BADL and 54 (19.4%) presented moderate–severe cognitive impairment. One hundred and eighty-eight (67.5%) had CFS scores ≥5 and 253 (91%) scored ≥2 on the ISAR scale. Higher levels of dependency for BADL and of frailty were recorded in women (Table 1).

### 3.1. The 3D/3D+ Tool

Two hundred and thirty-one patients (83.1%, 95% CI: 78.2–87.3) presented some degree of frailty according to the baseline component (3D): moderate in 94 (33.8%, 3D 2) and severe in 85 (30.6%, 3D 3). The prevalence of moderate–severe frailty was higher in women (Table 2).

One hundred and seventy-three patients (62.2%) presented moderate–severe functional dependence and 84 (30.2%) moderate–severe cognitive impairment. Two hundred and eight (74.8%) came to the hospital from their homes and of these, 30 (10.8%) lived alone.

The current component (3D+) presented alterations in 167 patients (60.1%, 95% CI: 54.1–65.9). In 104 patients (37.4%), the reason for consultation had been an acute functional impact, and in 66 (23.7%) delirium was detected on arrival at the ED. In 39 patients (14.0%), the physician considered that one or more of the prescribed medications may have been the trigger for the consultation and/or the functional and cognitive repercussions. In 55 cases (19.8%), home treatment was not feasible (Table 2).

### 3.2. Destination upon Discharge from the ED and Its Suitability

Regarding the destination upon discharge from the ED, 172 patients (61.9%) did not require admission to the acute care ward or the TU. Seventy-two (25.9%) were discharged to their habitual address. Discharge destination was considered to be appropriate in 96.4% (95% CI: 93.0–98.0) of cases.

### 3.3. Prognostic Accuracy of 3D/3D+ for Predicting Adverse Outcomes

Patients with alterations in delirium and functional decline in the 3D+ had a higher risk of presenting adverse outcomes at 30 days and higher short- and long-term mortality (Table 3).

The 3D+ (delirium-functional decline dimensions) showed better discriminative capacity than the CFS and the ISAR for predicting 30-day mortality (AUC = 0.80; 95% CI: 0.73–0.86), adverse outcome at 30 days (AUC = 0.66; 95% CI: 0.60–0.73) and six-month mortality (AUC = 0.71; 95% CI: 0.64–0.77, see Supplementary Table S2). Table 4 shows the prognostic performance parameters of the 3D+ (delirium-functional decline) for predicting adverse outcomes. The three scales presented similar NPVs for predicting 30-day mortality, 30-day adverse outcome, and six-month mortality, but the 3D+ (delirium–functional decline dimensions) showed the highest PPV. The discriminative capacity of the 3D/3D+ fell to values similar to those of the CFS in 12-month mortality. The NPV for long-term mortality decreased for all three scales.

## 4. Discussion

This study shows that the dynamic component (3D+) of the 3D/3D+ tool has a good predictive capacity for the prognosis of 30-day mortality in patients aged 75 or over with clinical complexity and frailty seen in the ED. Specifically, delirium and functional decline were the 3D+ dimensions that were independently associated with 30-day mortality. The appearance of delirium either alone or in combination with functional decline during acute illness increases the likelihood of presenting an adverse outcome at 30 days and mortality in either the short or the long term.

The CFS is one of the many instruments for rapid multidimensional geriatric assessment that have appeared recently in the literature. The CFS is a judgment-based tool that evaluates specific domains including comorbidity, functional capacity, and cognition to generate a frailty score ranging from 1 (very fit) to 9 (terminally ill) [16]. As a result of the COVID-19 pandemic, health personnel who are not experts in frailty frequently use the CFS to guide decisions regarding the allocation of healthcare resources [18,26,27]. The CFS has been shown to be a robust predictor of adverse outcomes in acute care settings [28,29] and has proved its usefulness in the ED setting [30,31,32,33]. Its predictive ability improves when the acuity of the illness is taken into account. Pulok et al. [8] described the strong association of CFS with all-cause 30-day mortality among ED patients referred to internal medicine, and stressed the influence of illness acuity on the association between frailty and mortality. The risk of 30-day mortality was highest among severely frail patients with high acuity (OR: 22.5; 95% CI: 9.35–62.12). Along the same lines, Nissen et al. [34] observed that the AUC for the combination of aggregated vital signs using the National Early Warning Score (NEWS) and frailty according to CFS was 0.86 (95% CI: 0.83–0.90), a figure that was significantly higher than the NEWS (AUC = 0.81; 95% CI = 0.77–0.85, *p* < 0.001) or the CFS alone (AUC = 0.82; 95% CI: 0.78–0.86, *p* < 0.001) and drew attention to an important clinical interaction between frailty and illness severity. There is also evidence of the ability of the CFS to predict long-term mortality. Rueegg et al. [35] showed the CFS to be a good predictor of mortality at one year (AUC = 0.77; 95% CI: 0.74–0.79) and superior to the Emergency Severity Index (ESI) (AUC = 0.70; 95% CI: 0.67–0.73; both models adjusted for age, sex, and presenting condition). In our study, the 3D+ (the delirium–functional decline dimensions) showed better discriminative capacity than the CFS for predicting mortality at 30 days, and similar values at 12 months.

The ISAR scale was developed by McCusker et al. [12] as a self-report screening tool to identify older people in hospital EDs at increased risk of six-month adverse health outcomes, including death, admission to a nursing home or long-term hospitalization, or a clinically significant decrease in functional capacity. The AUCs obtained were 0.70 (95% CI: 0.66–0.74) and 0.71 (0.66–0.76) in the development and validation samples, respectively. However, in two subsequent systematic reviews and meta-analyses [36] the overall sensitivity and specificity of the ISAR instrument ranged between 61% and 99% and 21% and 51%. The specificity values were particularly unsatisfactory. Both meta-analyses concluded that the ISAR is not sufficiently accurate to predict the likelihood of ED return visits, functional decline, hospital readmission, mortality or adverse outcomes in the short and medium term (1–12 months after the ED visit), and, therefore, lacks clinical utility. The sensitivity of the ISAR in our study ranged between 88% and 95% and its specificity between 8% and 11%. This low specificity for any of the adverse outcomes evaluated may be due to the fact that only 9% of patients obtained a score below 2 on the ISAR scale, a score that allows elderly patients to be safely discharged to their homes or to a nursing home. In contrast, based on the dynamic assessment (3D+) and clinical judgment, it was possible to discharge 25.9% of patients either to their homes or to a nursing facility.

This study had limitations such as its single-center and retrospective nature, and so caution is required when extrapolating the results to other settings. However, the data from the 3D/3D+ were recorded prospectively, since this tool was already part of the care practice of emergency professionals at our hospital during the study period. Patients were included consecutively and, in our view, comprised a representative sample of elderly patients with clinical complexity and frailty who are routinely seen in EDs. This stratum of the elderly population was selected because decisions regarding their care pose a particular challenge to emergency physicians, and because there is a strong likelihood that they will be assigned to conventional hospitalization in spite of the availability of other resources that may be more appropriate to their needs.

With regard to adverse outcomes and early mortality due to the impact of acute illness, two of the components of dynamic frailty—delirium and functional decline—were decisive in establishing the instrument’s prognostic validity, even more so than the baseline or static component (3D). Delirium is an acute confusional state that is extremely common among hospitalized elders and is strongly associated with poor short-term and long-term outcomes [37,38]. It is an independent marker for increased short- and long-term mortality among older medical inpatients during the 12 months after hospital admission [39,40,41,42]. Our study corroborates previous evidence on the association between delirium and mortality in elderly patients.

The dynamic condition of frailty, or acquired frailty, observed in our ED patients and its impact on short- and long-term adverse outcomes have also been described in patients admitted to acute care and intermediate care hospitals [43,44]. The transition between different frailty states may be modifiable and may improve prognosis [43]. Therefore, detecting these changes has clinical significance; it can reinforce decisions regarding the care resource indicated by the 3D/3D+ and can guide future decisions regarding therapeutic objectives.

The prognostic value of the 3D+ (the delirium–functional decline dimensions) in the context of ED can help decision making regarding the most appropriate care resource on ED discharge. Patients without delirium and/or without functional decline can be discharged more safely to their home or nursing home, thus avoiding hospital admission. Hospitalization is indicated when the short-term prognosis is unfavorable.

The 3D/3D+ is in line with other recently implemented innovative proposals for improving the care of elderly patients in Eds, such as the proposal of Megalla et al. [45] based on the 4M model—what matters, medication, mentation, and mobility—for providing care for older adults devised by the Institute for Healthcare Improvement (IHI) [46], or Puig-Campmany et al. [47] Program of Care for Frailty development. The EDs where these studies were carried out obtained Emergency Department Accreditation from the American College of Emergency Physicians.

The 3D/3D+ provides an optimal model of emergency care adapted for patients aged 75 years and older treated at EDs. It facilitates the training of ED professionals and helps to homogenize the care given to these patients. It stratifies the level of frailty (3D), quantifies the severity of patients’ acute problems (3D+) and contributes to decision making regarding the most appropriate care resource on ED discharge [17]. The 3D/3D+ allows effective, personalized and patient-centered care by identifying urgent medical, functional, psychological and social needs. As a novelty, the dynamic component (3D+) presents a good prognostic capacity for short-term mortality. The results presented here have practical applications, since the instrument can provide important information to guide the decision of whether to allow patients to return home, especially if there are solid community links, and it can help to establish future therapeutic objectives.

Further studies are now necessary to evaluate the external validity of the 3D/3D+ and its intra- and inter-observer reliability. These studies should also assess health outcomes such as the recovery of functional capacity and the experience of patients/family members.

## Figures and Tables

**Table 1:** Baseline characteristics: overall and according to sex.

	Total	Women	Men	*p*-Value
	N = 278	n = 166	n = 112	
Age [median (p25–p75)]	86.0 [83.0–90.0]	87.0 [83.0–91.8]	85.0 [81.8–89.0]	0.021
Gender (female)	166 (59.7)			NA
Time frame				0.523
07:01–14:00	120 (43.2)	71 (42.8)	49 (43.8)	
14:01–22:00	106 (38.1)	67 (40.4)	39 (34.8)	
22:01–07:00	52 (18.7)	28 (16.9)	24 (21.4)	
Triage level				0.222
II	47 (16.9)	24 (14.5)	23 (20.5)	
III	175 (62.9)	104 (62.7)	71 (63.4)	
IV	56 (20.1)	38 (22.9)	18 (16.1)	
Prior medical care at home (Yes)	119 (42.8)	76 (45.8)	43 (38.4)	0.222
Arrival at ED by ambulance (Yes)	223 (80.2)	141 (84.9)	82 (73.2)	0.016
PCC (Yes)	186 (66.9)	117 (70.5)	69 (61.6)	0.123
MACA (Yes)	36 (12.9)	18 (10.8)	18 (16.1)	0.203
Readmission (≥2 or more times/last year)	107 (38.9)	62 (37.6)	45 (40.9)	0.579
Reason for consultation				
Dyspnea	140 (50.4)	78 (47.0)	62 (55.4)	0.171
General malaise	40 (14.4)	30 (18.1)	10 (8.93)	0.033
Anemia	6 (2.16)	4 (2.41)	2 (1.79)	0.726
Fever	34 (12.2)	20 (12.0)	14 (12.5)	0.910
Chest pain	9 (3.24)	5 (3.01)	4 (3.57)	0.796
Abdominal pain	20 (7.19)	16 (9.64)	4 (3.57)	0.055
Locomotor pain	1 (0.36)	1 (0.60)	0 (0.00)	0.410
Falls	23 (8.27)	15 (9.04)	8 (7.14)	0.574
Neurological symptoms	38 (13.7)	22 (13.3)	16 (14.3)	0.806
Others	16 (5.76)	7 (4.22)	9 (8.04)	0.180
Barthel Index ^1^				0.041
Independent (90–100)	60 (23.9)	27 (18.1)	33 (32.3)	
Mild dependency (61–89)	51 (20.3)	29 (19.5)	22 (21.6)	
Moderate dependency (45–60)	63 (25.1)	41 (27.5)	22 (21.6)	
Severe dependency (<45)	77 (30.7)	52 (34.9)	25 (24.5)	
Pfeiffer Questionnaire ^2^				0.094
No cognitive impairment	128 (52.9)	67 (47.2)	61 (61.0)	
Mild cognitive impairment	67 (27.7)	43 (30.3)	24 (24.0)	
Moderate cognitive impairment	10 (4.1)	5 (3.5)	5 (5.0)	
Severe cognitive impairment	37 (15.3)	27 (19.0)	10 (10.0)	
Rockwood Clinical Frailty Scale				0.008
Fit (CFS 1)	3 (1.08)	0 (0.00)	3 (2.68)	
Well (CFS 2)	15 (5.40)	4 (2.41)	11 (9.82)	
Managing well (CFS 3)	28 (10.1)	13 (7.83)	15 (13.4)	
Vulnerable (CFS 4)	44 (15.8)	26 (15.7)	18 (16.1)	
Slightly frail (CFS 5)	32 (11.5)	18 (10.8)	14 (12.5)	
Moderately frail (CFS 6)	56 (20.1)	38 (22.9)	18 (16.1)	
Severely frail (CFS 7)	66 (23.7)	47 (28.3)	19 (17.0)	
Very severely frail (CFS 8)	30 (10.8)	19 (11.4)	11 (9.82)	
Terminally ill (CFS 9)	4 (1.4)	1 (0.60)	3 (2.68)	
ISAR scale				
≥2	253 (91.0)	157 (94.6)	96 (85.7)	0.011
≥3	209 (75.2)	137 (82.5)	72 (64.3)	<0.001
≥4	141 (50.7)	92 (55.4)	49 (43.8)	0.056
Diagnosis at ED discharge				0.524
Cardiac respiratory failure	32 (11.5)	21 (12.7)	11 (9.82)	
Lung respiratory failure	65 (23.4)	36 (21.7)	29 (25.9)	
Mixed respiratory failure	12 (4.3)	7 (4.22)	5 (4.46)	
Lung infection	36 (12.9)	18 (10.8)	18 (16.1)	
Abdominal infection	8 (2.9)	7 (4.22)	1 (0.89)	
Urinary infection	26 (9.4)	18 (10.8)	8 (7.14)	
Skin infection	2 (0.7)	1 (0.60)	1 (0.89)	
Fractures	3 (1.1)	3 (1.81)	0 (0.00)	
Stroke	12 (4.3)	7 (4.22)	5 (4.46)	
Others	82 (29.5)	48 (28.9)	34 (30.4)	
The patient or family prioritizes treatment at home (Yes)	121 (43.5)	73 (44.0)	48 (42.9)	0.854

ED: Emergency Department; PCC: *Pacient Crònic Complex* (chronic complex patient); MACA: *Malaltia Crònica Avançada* (patient with advanced chronic disease); ISAR: Identification of Seniors at Risk; NA: Not Applicable. ^1^ 27 missing values. ^2^ 36 missing values.

**Table 2:** The 3D/3D+ geriatric assessment tool.

Total N = 278		Women n = 166	Men n = 112	*p*-Value
3D Baseline component				
Difficulties with BADL, walking, or physical activity				<0.001
No (D0)	52 (18.7)	18 (10.8)	34 (30.4)	
Mild (D1)	53 (19.1)	28 (16.9)	25 (22.3)	
Moderate (D2)	96 (34.5)	66 (39.8)	30 (26.8)	
Severe (D3)	77 (27.7)	54 (32.5)	23 (20.5)	
Dementia/Cognitive impairment				0.142
No (D0)	139 (50.0)	75 (45.2)	64 (57.1)	
Mild (D1)	55 (19.8)	33 (19.9)	22 (19.6)	
Moderate (D2)	47 (16.9)	34 (20.5)	13 (11.6)	
Severe (D3)	37 (13.3)	24 (14.5)	13 (11.6)	
Dwelling				0.030
Lives in own home alone	30 (10.8)	18 (10.8)	12 (10.7)	
Lives in own home with family or caregiver	178 (64.0)	97 (58.4)	81 (72.3)	
Nursing home	70 (25.2)	51 (30.7)	19 (17.0)	
3D Baseline component				<0.001
No frailty (3D 0)	47 (16.9)	16 (9.6)	31 (27.7)	
Mild frailty (3D 1)	52 (18.7)	28 (16.9)	24 (21.4)	
Moderate frailty (3D 2)	94 (33.8)	65 (39.2)	29 (25.9)	
Advanced frailty (3D 3)	85 (30.6)	57 (34.3)	28 (25.0)	
3D+ Dynamic component				
Decline in BADL-IADL				0.530
No	116 (41.7)	65 (39.2)	51 (45.5)	
Yes, acute	104 (37.4)	66 (39.8)	38 (33.9)	
Yes, progressive	58 (20.9)	35 (21.1)	23 (20.5)	
Delirium				0.130
No	212 (76.3)	122 (73.5)	90 (80.4)	
Yes, hyperactive	17 (6.1)	14 (8.4)	3 (2.7)	
Yes, hypoactive	49 (17.6)	30 (18.1)	19 (17.0)	
Dwelling				0.572
Is 24 h treatment at home feasible? (No)	55 (19.8)	31 (18.7)	24 (21.4)	
Drugs				0.090
Might drugs (start/withdrawal) be a trigger of the presenting complaint?	39 (14.0)	29 (17.5)	10 (8.93)	
3D+ Dynamic component (impact of acute illness)				0.187
Impairment	167 (60.1)	105 (63.3)	62 (55.4)	
No impairment	111 (39.9)	61 (36.7)	50 (44.6)	

**Table 3:** Adverse 30-day outcomes and short- and long-term mortality.

		3D+ (Delirium)			3D+ (Delirium and Functional Decline)		
	N = 278	No Alteration n = 212	Alteration n = 66	*p*-Value	No Alteration n = 220	Alteration n = 58	*p*-Value
72 h ED returns	5 (1.8)	4 (1.9)	1 (1.5)	0.843	4 (1.8)	1 (1.7)	0.962
72 h hospital readmission	3 (1.1)	3 (1.4)	0 (0)	0.331	3 (1.6)	0 (0)	0.371
30-day ED returns	50 (18.0)	42 (19.8)	8 (12.1)	0.155	43 (19.6)	7 (12.1)	0.187
30-day hospital readmission	28 (10.1)	23 (10.9)	5 (7.6)	0.440	23 (10.5)	5 (8.6)	0.680
30-day mortality	54 (19.4)	21 (9.9)	33 (50.0)	<0.001	23 (10.5)	31 (53.5)	<0.001
30-day any adverse outcome	100 (36.0)	60 (28.3)	40 (60.6)	<0.001	63 (28.6)	37 (63.8)	<0.001
6-month mortality	86 (30.9)	45 (21.2)	41 (62.1)	<0.001	49 (22.2)	37 (63.8)	<0.001
12-month mortality	107 (38.5)	64 (30.2)	43 (65.2)	<0.001	69 (31.4)	38 (65.5)	<0.001

**Table 4:** Prognostic accuracy of 3D+, CFS and ISAR at cutoffs with corresponding 95% confidence intervals.

	Sensitivity (95% CI)	Specificity (95% CI)	PPV (95% CI)	NPV (95% CI)
30-day mortality				
3D+ (delirium)	0.61 (0.55–0.67)	0.85 (0.81–0.89)	0.50 (0.44–0.56)	0.90 (0.87–0.94)
3D+ (delirium and functional decline)	0.57 (0.52–0.63)	0.88 (0.84–0.92)	0.53 (0.48–0.59)	0.90 (0.86–0.93)
CFS (≥5)	0.81 (0.69–0.91)	0.36 (0.29–0.42)	0.23 (0.18–0.30)	0.89 (0.81–0.95)
CFS (≥7)	0.54 (0.40–0.67)	0.68 (0.62–0.74)	0.29 (0.20–0.39)	0.86 (0.80–0.91)
ISAR (≥2)	0.94 (0.85–0.99)	0.10 (0.06–0.14)	0.20 (0.15–0.26)	0.88 (0.69–0.97)
ISAR (≥3)	0.83 (0.71–0.92)	0.27 (0.21–0.33)	0.22 (0.16–0.28)	0.87 (0.77–0.94)
72 h ED returns				
3D+ (delirium)	0.20 (0.15–0.25)	0.76 (0.71–0.81)	0.02 (0.00–0.03)	0.98 (0.97–1.00)
3D+ (delirium and functional decline)	0.20 (0.15–0.25)	0.79 (0.74–0.84)	0.02 (0.00–0.04)	0.98 (0.97–1.00)
CFS (≥5)	1.00 (0.48–1.00)	0.33 (0.27–0.39)	0.03 (0.01–0.06)	1.00 (0.96–1.00)
CFS (≥7)	0.80 (0.28–0.99)	0.65 (0.59–0.70)	0.04 (0.01–0.10)	0.99 (0.97–1.00)
ISAR (≥2)	1.00 (0.48–1.00)	0.09 (0.06–0.13)	0.02 (0.01–0.05)	1.00 (0.86–1.00)
ISAR (≥3)	1.00 (0.48–1.00)	0.25 (0.20–0.31)	0.02 (0.01–0.05)	1.00 (0.95–1.00)
30-day ED returns				
3D+ (delirium)	0.16 (0.12–0.20)	0.75 (0.69–0.80)	0.12 (0.08–0.16)	0.80 (0.76–0.85)
3D+ (delirium and functional decline)	0.14 (0.10–0.18)	0.78 (0.73–0.83)	0.12 (0.08–0.16)	0.80 (0.76–0.85)
CFS (≥5)	0.66 (0.51–0.79)	0.32 (0.26–0.38)	0.18 (0.12–0.24)	0.81 (0.71–0.89)
CFS (≥7)	0.28 (0.16–0.42)	0.62 (0.56–0.69)	0.14 (0.08–0.22)	0.80 (0.73–0.85)
ISAR (≥2)	0.88 (0.76–0.95)	0.08 (0.05–0.13)	0.17 (0.13–0.23)	0.76 (0.55–0.91)
ISAR (≥3)	0.70 (0.55–0.82)	0.24 (0.18–0.30)	0.17 (0.12–0.23)	0.78 (0.67–0.87)
30-day any adverse outcome				
3D+ (delirium)	0.40 (0.34–0.46)	0.85 (0.81–0.90)	0.61 (0.55–0.66)	0.72 (0.66–0.77)
3D+ (delirium and functional decline)	0.37 (0.31–0.43)	0.88 (0.84–0.92)	0.64 (0.58–0.69)	0.71 (0.66–0.77)
CFS (≥5)	0.74 (0.64–0.82)	0.36 (0.29–0.43)	0.39 (0.32–0.47)	0.71 (0.61–0.80)
CFS (≥7)	0.41 (0.31–0.51)	0.67 (0.59–0.74)	0.41 (0.31–0.51)	0.67 (0.59–0.74)
ISAR (≥2)	0.92 (0.85–0.96)	0.10 (0.06–0.15)	0.36 (0.30–0.43)	0.68 (0.46–0.85)
ISAR (≥3)	0.78 (0.69–0.86)	0.26 (0.20–0.34)	0.37 (0.31–0.44)	0.68 (0.56–0.79)
6-month mortality				
3D+ (delirium)	0.48 (0.42–0.54)	0.87 (0.83–0.91)	0.62 (0.56–0.68)	0.79 (0.74–0.84)
3D+ (delirium and functional decline)	0.43 (0.37–0.49)	0.89 (0.85–0.93)	0.64 (0.58–0.69)	0.78 (0.73–0.83)
CFS (≥5)	0.81 (0.72–0.89)	0.39 (0.32–0.46)	0.37 (0.30–0.45)	0.82 (0.73–0.89)
CFS (≥7)	0.52 (0.41–0.63)	0.71 (0.64–0.78)	0.45 (0.35–0.55)	0.77 (0.70–0.83)
ISAR (≥2)	0.95 (0.89–0.99)	0.11 (0.07–0.16)	0.32 (0.27–0.39)	0.84 (0.64–0.95)
ISAR (≥3)	0.83 (0.73–0.90)	0.28 (0.22–0.35)	0.34 (0.28–0.41)	0.78 (0.67–0.87)
12-month mortality				
3D+ (delirium)	0.40 (0.34–0.46)	0.87 (0.83–0.91)	0.65 (0.60–0.71)	0.70 (0.64–0.75)
3D+ (delirium and functional decline)	0.36 (0.30–0.41)	0.88 (0.85–0.92)	0.66 (0.60–0.71)	0.69 (0.63–0.74)
CFS (≥5)	0.80 (0.72–0.87)	0.40 (0.33–0.48)	0.46 (0.38–0.53)	0.77 (0.67–0.85)
CFS (≥7)	0.51 (0.42–0.61)	0.74 (0.66–0.80)	0.55 (0.45–0.65)	0.71 (0.64–0.77)
ISAR (≥2)	0.93 (0.86–0.97)	0.10 (0.06–0.15)	0.39 (0.33–0.45)	0.68 (0.46–0.85)
ISAR (≥3)	0.79 (0.71–0.87)	0.27 (0.21–0.35)	0.41 (0.34–0.48)	0.68 (0.56–0.79)

## Data Availability

The data presented in this study are available on request from the corresponding author.

## Supplementary Materials

The following supporting information can be downloaded at: https://www.mdpi.com/article/10.3390/jcm12206469/s1, Table S1: Association between 3D/3D+ and 30-day mortality. Adjusted odds ratio (aOR) and corresponding statistical significance according to multivariable logistic regression models; Table S2: Discriminative ability of 3D+, CFS and ISAR. Area under the curve ROC (95% CI).
