# Supplementary material for: Prognostic Value of a New Tool (the 3D/3D+) for Predicting 30-Day Mortality in Emergency Department Patients Aged 75 Years and Older
Source: J Clin Med. 2023 Oct 11;12(20):6469. doi: 10.3390/jcm12206469 (PMC10607455; doi:10.3390/jcm12206469)
Supplement: Supplementary file 1 [file jcm-12-06469-s001.zip › jcm-2616383-supplementary.pdf]

Supplementary Materials

Table S1. Association between 3D/3D+ and 30-day mortality. Adjusted odds ratio (aOR) and corresponding statistical significance according to multivariable logistic regression models.

	**Model 1 aOR (95% CI)**	**p-value**	**Model 2 aOR (95% CI)**	**p-value**	**Model 3 aOR (95% CI)**	**p-value**	**Model 4 aOR (95% CI)**
**3D Baseline component**					-		-
No frailty (3D 0)	1		1				
Mild frailty (3D 1)	0.89 (0.24-3.30)	0.866	0.72 (0.17-3.00)	0.655			
Moderate frailty (3D 2)	2.57 (0.90-7.28)	0.076	1.48 (0.47-4.67)	0.503			
Severe frailty (3D 3)	2.93 (1.03-8.35)	0.044	1.27 (0.39-4.20)	0.691			
**3D+ Current component**							
**Functional decline**							
No			1		1		1
Yes, acute			4.49 (1.65-12.19)	0.003	4.64 (1.72-12.51)	0.002	4.25 (1.59-11.37)
Yes, progressive			5.00 (1.73-14.49)	0.003	5.19 (1.81-14.90)	0.002	5.01 (1.75-14.31)
**Delirium**							
No			1		1		1
Yes, hyperactive			6.06 (1.92-19.11)	0.002	6.41 (2.09-19.67)	0.001	6.01 (2.01-17.99)
Yes, hypoactive			6.64 (2.98-14.80)	<0.001	6.97 (3.19-15.24)	<0.001	6.44 (3.01-13.79)
**24-hour treatment at home is feasible**							-
Yes			1		1		
No			0.60 (0.24-1.52)	0.283	0.58 (0.20-1.44)	0.237	
**Medication explains the reason for consultation**							-
No			1		1		
Yes			0.58 (0.21-1.61)	0.299	0.54 (0.20-1.44)	0.220	
Area under the curve ROC (95% CI)	**0.62 (0.54-0.69)**		**0.82 (0.76-0.88)**		**0.81 (0.75-0.87)**		**0.80 (0.74-0.87)**

Table S2. Discriminative ability of 3D+, CFS and ISAR. Area under the curve ROC (95%CI).

	**AUC (95%CI)**
**Return to ED within 72 hours**	
3D+ (delirium - functional decline)	0.64 (0.51-0.77)
CFS	0.72 (0.54-0.90)
ISAR	0.52 (0.39-0.65)
**Return to ED within 30 days**	
3D+ (delirium - functional decline)	0.45 (0.37-0.53)
CFS	0.45 (0.36-0.54)
ISAR	0.46 (0.37-0.55)
**30-day mortality**	
3D+ (delirium - functional decline)	0.80 (0.73-0.86)
CFS	0.67 (0.59-0.76)
ISAR	0.58 (0.50-0.67)
**Some adverse outcome at 30 days**	
3D+ (delirium - functional decline)	0.66 (0.60-0.73)
CFS	0.58 (0.51-0.65)
ISAR	0.54 (0.47-0.61)
**6-month mortality**	
3+ (delirium - functional decline)	0.71 (0.64-0.77)
CFS	0.67 (0.61-0.75)
ISAR	0.61 (0.54-0.68)
**12-month mortality**	
3D+ (delirium - functional decline)	0.66 (0.60-0.72)
CFS	0.69 (0.63-0.75)
ISAR	0.60 (0.53-0.67)

AUC: Area under the curve ROC (95%CI)
